# Endoscopic visualization of the inferior alveolar nerve associated with somatosensory changes after impacted mandibular third molar extraction

**DOI:** 10.1007/s10266-023-00788-y

**Published:** 2023-02-11

**Authors:** Jun-Qi Jiang, Yan-Feng Kang, Ke-Nan Chen, Nian-Hui Cui, Zi-Yu Yan, Chuan-Bin Guo, En-Bo Wang, Xiang-Liang Xu

**Affiliations:** 1 Department of Oral and Maxillofacial Surgery, Peking University School and Hospital of Stomatology and National Center of Stomatology and National Clinical Research Center for Oral Diseases and National Engineering Research Center of Oral Biomaterials and Digital Medical Devices and Beijing Key Laboratory of Digital Stomatology and Research Center of Engineering and Technology for Computerized Dentistry Ministry of Health and NMPA Key Laboratory for Dental Materials, No.22 Zhongguancun South Avenue, Haidian District Beijing, 100081 People’s Republic of China; 2 Department of Prosthodontics Center for Oral Functional Diagnosis, Treatment and Research, Peking University School and Hospital of Stomatology, Beijing, China

**Keywords:** Endoscopy, Impacted lower third molars, Quantitative sensory testing, Inferior alveolar nerve, Tooth extraction

## Abstract

The aim of this study is to assess the relationship between somatosensory functional changes and inferior alveolar nerve (IAN) exposure after impacted mandibular third molars (M3M) removal. We recruited 35 patients who underwent impacted M3M extraction near the IAN. The M3Ms were extracted by combined endoscopy, piezosurgery, and contra-angle high-speed turbine handpiece. All IAN canal perforations and exposed regions were recorded and measured by endoscopy after extraction and on cone-beam computed tomography (CBCT) images before extraction. The patients were followed up 1, 7, and 35 days after surgery. A standardized quantitative sensory testing (QST) battery was performed on the lower lip skin. All of 35 cases had exposed IAN on CBCT images, 5 of which had no exposed IAN under endoscopy. For the other 30 cases, the endoscopy-measured IAN length and width were shorter than the CBCT measurements (*P* < 0.001). The warm and mechanical detection thresholds (MDT) on the operation side were significantly higher than the contralateral side after surgery (*P* < 0.05). Thermal sensory limen, MDT, and cold pain threshold were strongly correlated with the exposed IAN length and MDT also with the exposed IAN width one day after surgery. In conclusion, it was found that not all exposed IAN in CBCT images were real exposure after surgery. The intraoperative exposed IAN endoscopic measurements were smaller than by CBCT and strongly correlated with some QST parameters.

## Introduction

Extraction of impacted mandibular third molars (M3M) is one of the most common oral and maxillofacial surgeries. The operation is technically difficult because of the position and morphology of the third molars, their relationship with adjacent teeth, and more. Frequent postoperative complications include facial swelling, pain, trismus, hemorrhage, and inferior alveolar nerve injury (IANI) [1, 2]. The reported IANI incidence is 0.41–8.10% for temporary injuries and 0.01–3.60% for permanent ones, which depending on the patient can contain a serious problem [3]. The main IANI might include dysesthesia, anesthesia, paresthesia, or hyperalgesia of the skin, mucous membrane and teeth innervated by the inferior alveolar nerve (IAN). Even minor changes could influence the patient’s physical and psychological well-being [4]. IANI has many risk factors including age, sex, cortical defect size of the inferior alveolar canal on maxillofacial CT images and the surgical technique of these, the cortical defect size could be a critical factor [5].

With the development of endoscopic technology, surgical sites visualization, including those difficulties to accesses, has improved [6]. The technology allows operative field magnification and digital recording and is widely applied in orofacial surgery. As a magnifying optical tool, it provided more adequate and direct insight for those complex cases with difficult access than common magnifying glasses. Meanwhile, endoscopy could record the images during the surgery, which could not be achieved by common magnifying glasses and loupes. In 2014, Engelke was the first to use endoscopy to remove impacted M3M suggesting that this method may reduce postoperative morbidity without increasing the risk of IANI [7]. Several studies claimed to successfully remove the M3M residual roots in the maxillofacial space by endoscopy and that the procedure was safe and fast [8]. Besides, endoscopy can directly visualize the IAN and other anatomic structures. Bonte et al. used endoscopy to observe the IAN after impacted M3M removal, something reported only in a single observational case without mentioning the relationship between the exposed IAN and IANI [9]. Although sighting an exposed intact IAN bundle during third molar surgery indicated a 20% risk of paresthesia, the outcome mostly depended on the surgeon’s experience [10]. Furthermore, maxillofacial surgeons have stressed the importance of adequate IAN visualization during surgery to achieve more predictable treatment outcomes and fewer postoperative complications [11].

Piezosurgery was introduced as an innovative system for an impacted tooth removal. The major advantages of piezosurgery included soft tissue protection, optimal surgical field of vision and reduced blood loss [12]. Some studies have demonstrated that piezosurgery had fewer postoperative complications, such as pain and trismus, than the rotary system. However, the evidence was insufficient to determine whether piezosurgery could reduce neurological complications and postoperative swelling [13, 14].

The German Research Network on Neuropathic Pain established a standardized quantitative sensory testing (QST) protocol to evaluate the thermal and mechanical somatosensory functions [15, 16]. Yan et al. confirmed that QST was sensitive enough to detect somatosensory abnormalities in IAN function [17]. With 13 thermal and mechanical testing procedures, QST can help perceive sensory signs that indicate possible central or peripheral sensitization. QST can quantitatively measure the somatosensory sensitivity changes related to IANI [16].

Impacted M3M was extracted in this study using a high-speed turbine handpiece and piezosurgery (dual-power) under endoscopic visualization. We aimed to observe and measure the exposure range of the IAN using endoscopy, compared these with measurements made on CBCT images, and assessed their association with the IAN canal cortical integrity. Furthermore, we first used a standardized QST protocol to assess the association between somatosensory functional changes and IAN exposure after endoscopy-assisted M3M extraction.

## Patients and methods

### Participants

This study was performed in line with the principles of the Declaration of Helsinki. The biomedical ethics committee of Peking University Hospital of Stomatology approved this study (PKUSSIRB-201949142) and written informed consent was obtained from all participants. We recruited 35 Chinese patients with an impacted M3M to be extracted at the Department of Oral and Maxillofacial Surgery of Peking University School and Hospital of Stomatology, China, between May and December 2020. All of patients have no underlying disease. The CBCT images were acquired with 3D Accuitomo (J Morita Mfg. Corp., Kyoto, Japan). The scan parameters were as follows: tube potential of 85–90kVp, tube current of 5 mA, field of view (FOV) of 6 cm × 6 cm, and a voxel size of 0.125 mm. Slice thickness and interval were both set at 0.2 mm.

Inclusion criteria:

1. Aged 18–45 years with no sex restrictions;
2. The M3M was completely mesially or horizontally impacted;
3. At least one or the M3M roots directly oppressed or encircled the IAN based on preoperative CBCT, and there was no observable bone by the naked eyes between the roots and IAN, which was defined as the exposed IAN on CBCT.

Exclusion criteria:

1. During the acute inflammatory stage or/and any other surgery contraindication;
2. Accompanied by a nervous system disease or taking drugs that affect the nervous system function during the clinical trial;
3. Preoperative sensory dysfunction of the lower lip or tongue;
4. CBCT showed mandibular second molar distal root absorption;

### Imaging variables

**Fig. 1:**
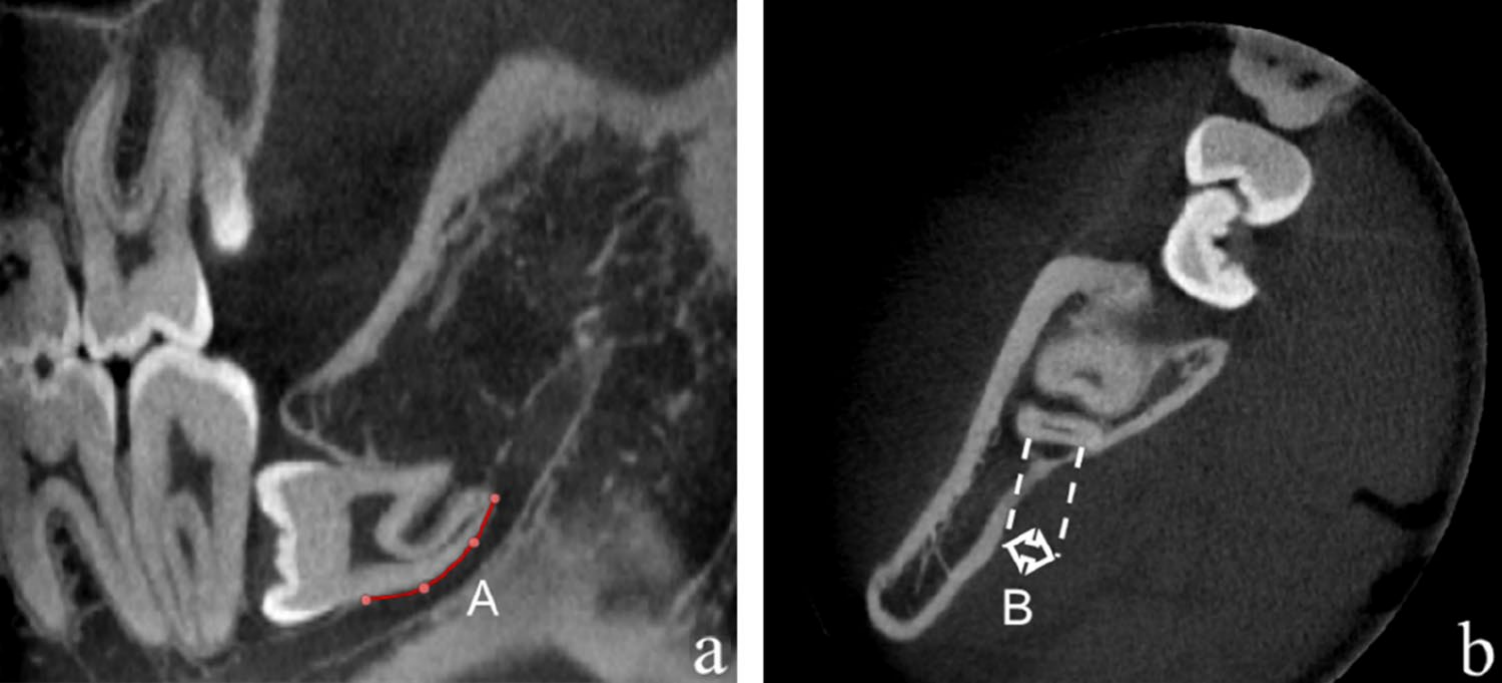
Measurement of the cortical perforation (mm). When the inferior alveolar canal (IAC) was located underneath the roots, the sagittal plane was adjusted to transect the long axis of the roots and the adjacent IAC. **a** A indicates the largest cortical defect length; **b** B indicates the largest cortical defect width

The predictor variables were the perforation length and width in the IAN canal in the third molar region. These were measured in the picture archiving communication system (Carestream Health, Inc, Rochester, NY). The XYZ axes of the CBCT multiplanar reformation were adjusted to achieve a cross-section with the long axis of the IAN passing along the M3M completely. The cortical defect length and width were defined as the longest exposed distances measured along the long axis and cross section of IAN (Fig. 1).

### Surgical procedure

**Fig. 2:**
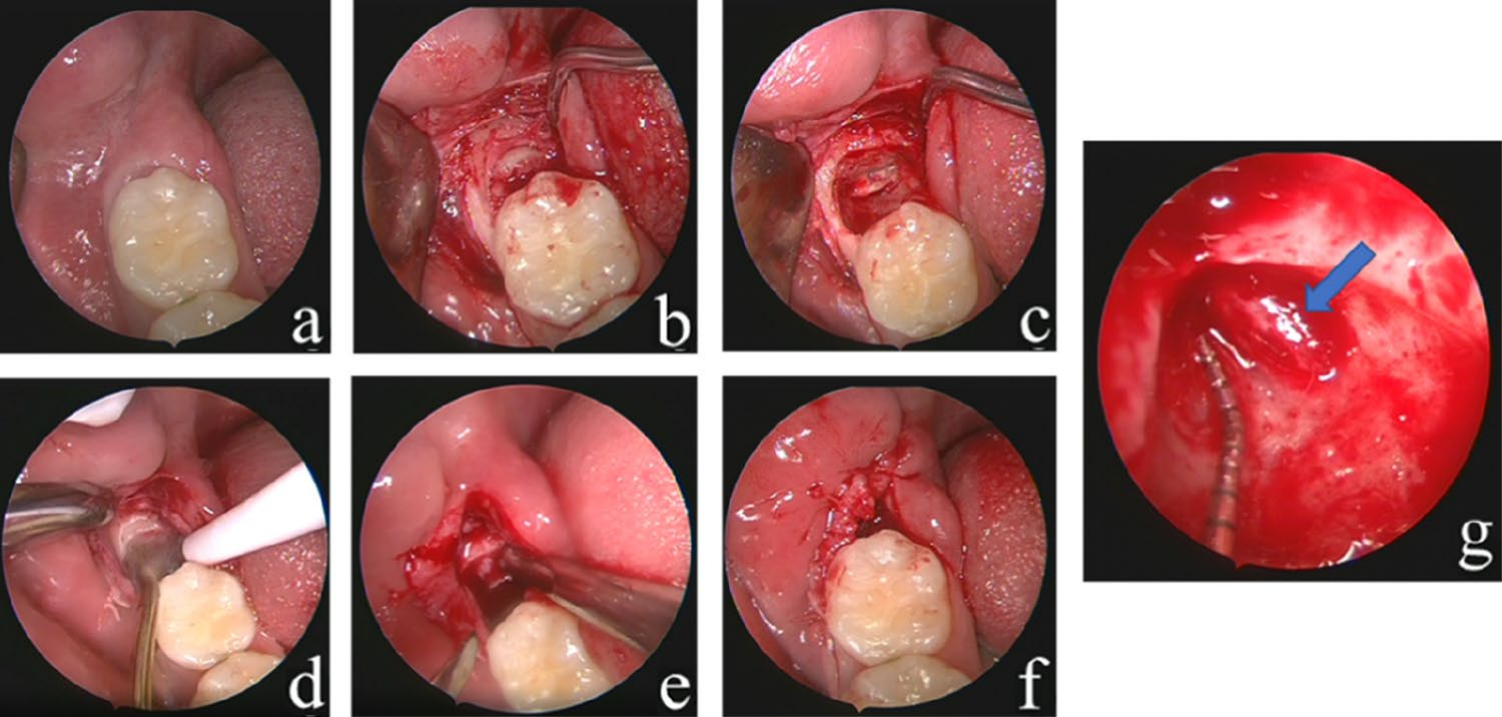
Protocol of tooth extraction by endoscopic surgery. **a** Preoperative images; **b** Flapped and exposed; **c** The mesial crown removed; **d** The roots are divided by piezosurgery; **e** The roots are elevated; **f** Wound suturing; **g** IAN exposure is recorded (The blue arrow points at IAN). *Pos 1* postoperative day 1, *Pos 7* postoperative day 7, *Pos 35* postoperative day 35

Surgery was performed under local anesthesia (4% articaine with 1:100,000 epinephrine). After making an extraction incision between the mandibular second molar and the mesial root of the mandibular wisdom tooth and separating the mucoperiosteum, we removed the bone, segmented the tooth and released the proximal crown resistance using the contra-angle high-speed turbine handpiece. Endoscope was used to check the part of crown next to the distal root surface of lower second molar which was difficult to check by naked eye. A Storz Hopkins endoscope (Karl Storz, Tuttlingen, Germany; Cat. No. 20223020) with 30° or 70° view angle and 4.0 mm diameter was used during the surgeries. After the crown was removed, the surgeon held the endoscope in the left hand and the piezosurgery in the right hand. The endoscope was placed on the buccal or lingual side, away from the surgical field. Under real-time endoscopic visualization, the roots were segmented and the bone next to the roots was partially removed by piezosurgery. The residual root was extracted by minimally invasive elevator under endoscopic visualization to observe the IAN condition and protect it. The final examination of the IAN was performed and recorded under endoscopic visualization. The IAN exposed region was measured using the scale of a prebent periodontal probe and the vascular nerve bundle pulsation was observed (Fig. 2). The socket was rinsed with physiological saline, and the incision was closed with absorbable sutures. All patients received amoxicillin 500 mg 3 times daily for 3 days.

### Observation of IAN and clinical evaluation variables

The exposed range of the IAN under endoscopy was recorded by the longest and widest distances (mm).

Neurovascular bundle pulsation under endoscopy was observed or not.

Operating time was recorded from incision to extraction of the tooth completely.

The patients indicated the pain level using a 10-point visual analog scale (VAS).

The degree of mouth opening was measured as the distance between the mesio-incisal edges of the upper and lower right central incisors at maximum mouth opening.

Facial swelling evaluation was performed using horizontal and vertical guides with a flexible tape on four reference points: tragus, outer corner of the mouth, outer canthus of the eye, and the mandible angle. Swelling (%) = (Postoperative value − Preoperative value)/Preoperative value × 100% [18];

Postoperative complications, such as hemorrhage, numbness of the lower lip, dry socket, and others, were recorded.

### Quantitative sensory testing

QST of the IAN was applied four times to the skin over the mental foramina on the operation and contralateral sides one week before surgery and one, seven, and 35 days after surgery. All QST measures were performed in a quiet room at 21–23℃. The QST consisted of seven subtests, measuring 13 thermal and mechanical parameters. Testing comprised cold detection threshold (CDT), warm detection threshold (WDT), thermal sensory limen (TSL), paradoxical heat sensation (PHS), cold pain threshold (CPT), heat pain threshold (HPT), mechanical detection threshold (MDT), mechanical pain threshold (MPT), dynamic mechanical allodynia (DMA), mechanical pain sensitivity (MPS), wind-up ratio (MUR), vibration detection threshold (VDT), and pressure pain threshold (PPT). The assessment method was as described by Yan et al. [17].

### Statistical analysis

The statistical analysis was performed using IBM SPSS Statistics for Windows Version 24.0 (IBM Corp., Armonk, NY, USA). Continuous variables are represented as mean ± standard deviation (SD). The IAN perforation length and width measurements were compared between the CT and endoscopy using the paired-samples *t* test. The QST results were log-transformed before analysis [19, 20].

Each individual QST variable datapoint was Z-transformed based on the reference data: *Z* = (value_operation side_ − mean_contralateral side_)/SD_contralateral side_ [21]. Since the right-left mean difference was zero, the 95% confidence interval (CI) of the relative reference data was calculated as zero (1.96 ± SD). Z-scores > 1.96 or < –1.96 indicated values outside of the 95% CI of the reference group data. Such values were considered abnormalities [15, 20]. We measured the operation and contralateral sides to greatly minimize the influence of patient-related subjective factors. One-way repeated measure analysis of variance followed by Bonferroni analyses was used to compare the groups of different time points. Correlations between the QST parameters and the length and width of exposed IAN were assessed using Pearson’s correlation coefficient. The significance level was set at *P* < 0.05.

## Results

This study included 35 patients, 20 females and 15 males, aged 30.5 ± 5.7 (range 19–43) years. No significant age or gender differences were detected in them. A total of 35 M3M extracted surgeries were performed. All operations were completed with no intraoperative complications, such as bleeding or root fragment displacement.

### Observation of the IAN

All of 35 cases had exposed IAN on CBCT images. However, 5 of these cases had no exposed IAN under endoscopy, with the length (7.89 ± 3.57 mm) and width (2.18 ± 0.29 mm) measured by CBCT. For the other 30 cases, the IAN length (5.43 ± 1.87 mm) and width (2.20 ± 0.85 mm) measured by endoscopy after extraction were shorter than the respective CBCT measurements (8.95 ± 2.07 mm and 3.35 ± 1.13 mm; *P* < 0.001 for both), with respective average shrinkage values of 40.9 ± 22.4% and 21.0 ± 17.8% (Table 1). The neurovascular bundle pulsation could be observed by endoscopy in 13 of the 35 cases during the surgery.

**Table 1:** Comparison between the CBCT and endoscopic measurements of the exposed IAN length and width

	Length(mm)	Width(mm)
Endoscopy	5.43 ± 1.87	2.20 ± 0.85
CBCT	8.95 ± 2.07	3.31 ± 1.13
*P*	0.000*	0.000*

Values are presented as means ± standard deviations

The two measuring techniques were compared by paired-samples *t* test

*CBCT* cone-beam computed tomography, *IAN* inferior alveolar nerve

*P < 0.05

### QST results

#### Comparison of preoperative and postoperative QST measurements

No PHS and DMA were found in this study. All other Z-transformed QST parameters at various follow-up times are presented in Table 2, in the range between –1.96 and 1.96. The one-way repeated measure analysis of variance results before surgery, one, seven and 35 days after surgery are presented in Table 3. There were no significant differences comparing the preoperative and postoperative values. WDT on one day after surgery differed significantly from the values on seven and 35 after surgery. HPT differed significantly between seven and 35 days after surgery. The remaining comparisons found no significant differences.

**Table 2:** The Z-transformed QST parameters’ values at various assessment time points

QST	Pre	Pos 1	Pos 7	Pos 35
CDT	− 0.079 ± 0.951	− 0.074 ± 0.974	− 0.159 ± 0.910	− 0.158 ± 1.372
WDT	− 0.151 ± 0.916	0.184 ± 0.731	− 0.191 ± 0.913	− 0.283 ± 0.938
TSL	− 0.054 ± 1.032	0.079 ± 0.878	0.053 ± 1.128	− 0.404 ± 1.237
HPT	0.195 ± 0.937	0.156 ± 1.101	0.043 ± 0.936	0.407 ± 1.031
CPT	− 0.089 ± 0.836	− 0.105 ± 0.838	− 1.406 ± 1.363	− 0.042 ± 1.039
MDT	− 0.049 ± 0.905	0.240 ± 1.091	0.231 ± 1.127	0.065 ± 0.964
MPT	− 0.003 ± 0.915	− 0.152 ± 0.988	− 0.313 ± 1.047	0.021 ± 0.871
MPS	− 0.026 ± 1.028	0.133 ± 1.059	0.225 ± 1.058	0.051 ± 0.927
MUR	− 0.095 ± 1.017	0.085 ± 1.063	0.169 ± 0.982	− 0.059 ± 0.912
VDT	− 0.017 ± 1.025	− 0.327 ± 1.025	− 0.225 ± 1.108	− 0.018 ± 1.104
PPT	0.013 ± 0.967	− 0.306 ± 1.403	− 0.137 ± 0.946	− 0.021 ± 1.046

Values are presented as means ± standard deviation

*QST* quantitative sensory testing, *CDT* cold detection threshold, *WDT* warm detection threshold, *TSL* thermal sensory limen, *PHS* paradoxical heat sensation, *CPT* cold pain threshold, *HPT* heat pain threshold, *MDT* mechanical detection threshold, *MPT* mechanical pain threshold, *DMA* dynamic mechanical allodynia, *MPS* mechanical pain sensitivity, *WUR* wind-up ratio, *VDT* vibration detection threshold, *PPT* pressure pain threshold, *Pre* preoperative, *Pos 1* postoperative day 1, *Pos 7* postoperative day 7, *Pos 35* postoperative day 35

**Table 3:** Between-time point comparisons of the QST parameters (*P* value)

QST	Pre versus Pos 1	Pre versus Pos 7	Pre versus Pos 35	Pro 1 versus Pos 7	Pro 1 versus Pos 35	Pro 7 versus Pos 35
CDT	1.000	1.000	1.000	1.000	1.000	1.000
WDT	0.104	1.000	1.000	0.022*	0.026*	1.000
TSL	1.000	1.000	0.349	1.000	0.166	0.317
HPT	1.000	1.000	1.000	1.000	0.469	0.021*
CPT	1.000	1.000	1.000	1.000	1.000	1.000
MDT	0.056	0.261	1.000	1.000	1.000	1.000
MPT	1.000	0.321	1.000	0.773	1.000	0.125
MPS	1.000	0.518	1.000	1.000	1.000	0.563
MUR	1.000	0.884	1.000	1.000	1.000	0.794
VDT	0.415	1.000	1.000	0.347	1.000	1.000
PPT	0.447	1.000	1.000	1.000	1.000	1.000

All comparisons were made by One-way repeated measure analysis of variance followed by Bonferroni analyses

Presented are the *P* values

*QST* quantitative sensory testing, *CDT* cold detection threshold, *WDT* warm detection threshold, *TSL* thermal sensory limen, *PHS* paradoxical heat sensation, *CPT* cold pain threshold, *HPT* heat pain threshold, *MDT* mechanical detection threshold, *MPT* mechanical pain threshold, *DMA* dynamic mechanical allodynia, *MPS* mechanical pain sensitivity, *WUR* wind-up ratio, *VDT* vibration detection threshold, *PPT* pressure pain threshold, *Pre* preoperative, *Pos 1* postoperative day 1, *Pos 7* postoperative day 7, *Pos 35* postoperative day 35

*P < 0.05

#### Relationship between QST measurements and the endoscopy measurements of the exposed IAN

The length of the exposed IAN under endoscopy was strongly correlated with TSL (*r* = 0.345, *P* = 0.042), CPT (*r* = –0.459, *P* = 0.006), and MDT (*r* = 0.398, *P* = 0.018) on one day after surgery. The width of the exposed IAN under endoscopy was strongly correlated with WDT(*r* = 0.337, *P* = 0.048) on one day after surgery but not seven and 35 days after surgery. The correlations between the other QST parameters and the range of exposed IAN were insignificant on one, seven, and 35 days after surgery (Table 4).

**Table 4:** Pearson’s correlation coefficient for the association between the QST data and the length and width of the exposed IAN

QST	IAN Length			IAN Width		
	Pos 1	Pos 7	Pos35	Pos 1	Pos 7	Pos35
CDT	0.15	0.131	0.178	− 0.065	0.086	0.175
WDT	0.189	0.009	0.048	0.3	0.119	0.13
TSL	0.345*	− 0.054	− 0.051	0.263	− 0.067	0.062
CPT	0.184	0.116	− 0.059	0.161	0.203	− 0.148
HPT	− 0.459*	− 0.171	− 0.03	− 0.258	− 0.132	− 0.067
MDT	0.398*	0.176	0.202	0.337*	0.118	0.139
MPT	− 0.089	0.294	− 0.083	0.104	0.273	− 0.003
MPS	0.02	0.078	− 0.169	− 0.075	0.041	− 0.239
WUR	− 0.051	0.117	− 0.003	− 0.215	0.257	− 0.004
VDT	− 0.133	0.153	− 0.167	− 0.011	0.088	− 0.132
PPT	0.153	− 0.057	0.061	0.211	0.115	0.083

Presented are the correlation values (*r* values)

*IAN* inferior alveolar nerve, *QST* quantitative sensory testing, *CDT* cold detection threshold, *WDT* warm detection threshold, *TSL* thermal sensory limen, *PHS* paradoxical heat sensation, *CPT* cold pain threshold, *HPT* heat pain threshold, *MDT* mechanical detection threshold, *MPT* mechanical pain threshold, *DMA* dynamic mechanical allodynia, *MPS* mechanical pain sensitivity, *WUR* wind-up ratio, *VDT* vibration detection threshold, *PPT* pressure pain threshold, *Pre* preoperative, *Pos 1* postoperative day 1, *Pos 7* postoperative day 7, *Pos 35* postoperative day 35

*P < 0.05

### Descriptive analysis of two cases with nerve injury after surgery

**Fig. 3:**
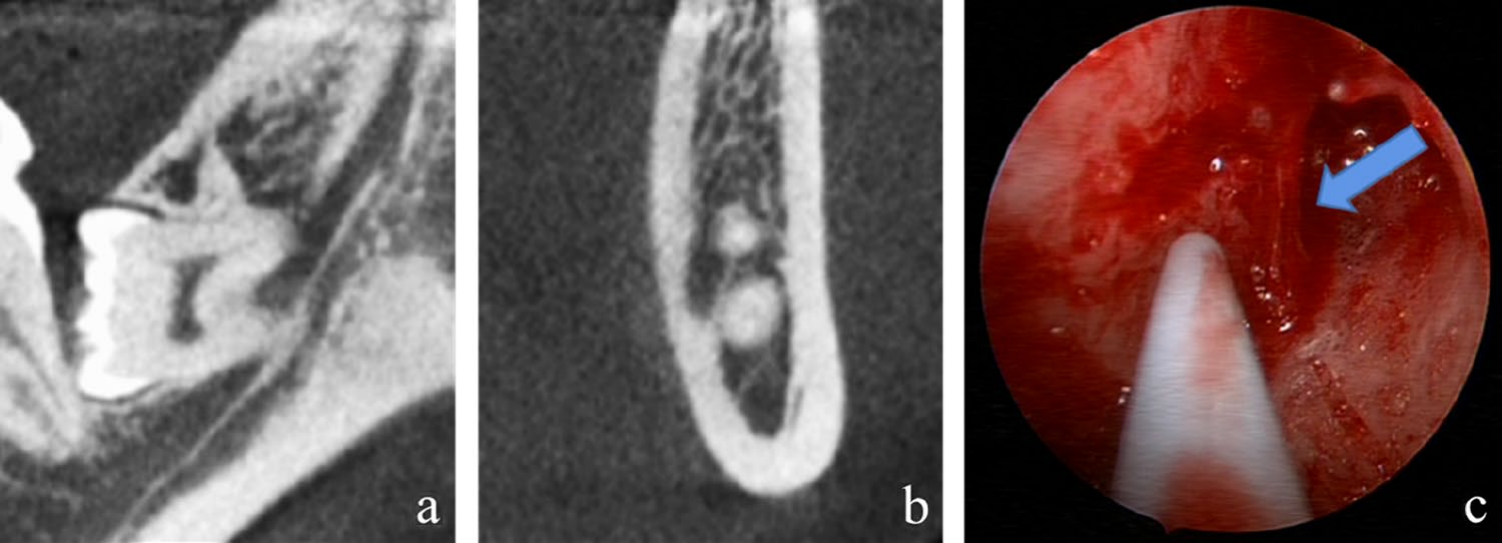
**a** and **b** Show the position of the inferior alveolar canal (IAC) to the third molar roots on CBCT images of a patient. The IAC position was categorized into inter-radicular position. **c** Shows endoscopic view of a possible injured IAN indicated by blue arrow

Two patients complained of lower lip numbness that recovered on ten days and two months after surgery, respectively. They were both females, one was 33 years old and the other was 26 years old. The QST findings differed between the two cases. The QST values in one case were in the normal ranges. However, the WDT and TSL on seven days after surgery and the TSL on 35 days after surgery of the other case were abnormal (Table 5). The coronal and cross-sectional CBCT images of this patient are shown in Fig. 3. There were some IANI risk factors in these two cases, including the lingual and inter-radicular IAN position respective to the M3M, and multiple roots with perforated mandibular canals [5]. The injured IANs were recorded under endoscopy which showed rough (Fig. 3).

**Table 5:** The Z-transformed QST parameters’ values of the two patients with nerve injury after surgery

QST	Case 1			Case 2		
	Pos 1	Pos 7	Pos 35	Pos 1	Pos 7	Pos 35
CDT	0.68	− 0.57	− 1.03	0.9	1.03	− 0.68
WDT	− 0.22	− 1.18	0.48	1.9	1.97*	0.5
TSL	1.8	− 0.98	− 0.28	0.6	2.76*	− 2*
HPT	− 0.81	− 0.35	0.05	− 0.32	0.59	− 0.17
CPT	0.22	0.4	0.21	0.2	0.4	0.49
MDT	0.51	0.04	0.12	0.51	0.04	0.12
MPT	− 0.43	0.31	0.5	− 0.21	− 0.98	− 0.08
MPS	1.1	0.7	0.86	0.07	0.47	0.15
MUR	0.96	0.13	1.13	0.25	0.64	1.22
VDT	− 0.67	0.28	0.35	0.15	− 0.23	0.35
PPT	− 0.9	− 0.73	− 0.33	0.46	− 0.06	0.22

*QST* quantitative sensory testing, *CDT* cold detection threshold, *WDT* warm detection threshold, *TSL* thermal sensory limen, *PHS* paradoxical heat sensation, *CPT* cold pain threshold, *HPT* heat pain threshold, *MDT* mechanical detection threshold, *MPT* mechanical pain threshold, *DMA* dynamic mechanical allodynia, *MPS* mechanical pain sensitivity, *WUR* wind-up ratio, *VDT* vibration detection threshold, *PPT* pressure pain threshold, *Pre* preoperative, *Pos 1* postoperative day 1, *Pos 7* postoperative day 7, *Pos 35* postoperative day 35

*P < 0.05

### Clinical assessments

The mean operation time was 25.7 ± 12.8 (range 10–58) minutes. Two patients complained of lower lip numbness that recovered on 10 days after surgery in one and two months after surgery in the other (Paresthesia rate was 6.7%). No postoperative complications such as infection or dry socket were recorded.

The mean pain VAS on one and seven days after surgery were 2.40 ± 1.75 and 0.91 ± 1.60, respectively. The mean maximal mouth opening decreased by 5.43 and 5.06 mm on one and seven days after surgery, respectively, and increased by 1.54 mm on 35 days after surgery. All differences from the baseline value were statistically significant. The mean swelling percent was 0.83 ± 2.04% on one day after surgery, 0.19 ± 1.91% on seven days after surgery (*P* = 0.108), and –0.16 ± 2.04% on 35 days after surgery (*P* = 0.030). The swelling percent on seven and 35 days after surgery were both smaller than that of one day after surgery, the latter was statistically different (Tables 6 and 7).

**Table 6:** Mouth opening comparison between before and after surgery

Assessment time	Mouth opening (mm)	*P* value
Pre	44.83 ± 5.65	
Pos 1	39.40 ± 9.74	0.000*
Pos 7	39.77 ± 10.72	0.001*
Pos 35	46.37 ± 6.89	0.019*

Values are presented as means ± standard deviations

The measurements on Pos 1, Pos 7, and Pos 35 were compared by paired-samples *t* test to the Pre

*Pre* preoperative, *Pos 1* postoperative day 1, *Pos 7* postoperative day 7, *Pos 35* postoperative day 35

*P < 0.05

**Table 7:** Comparisons of the swelling rate between before and after surgery

Assessment time	Mean swelling difference ± SD (%)	*P* value
Pos 1	0.83 ± 2.04	
Pos 7	0.19 ± 1.91	0.108
Pos 35	− 0.16 ± 2.04	0.030*

Values are presented as mean differences ± standard deviations (SDs)

The measurements on Pos 7, and Pos 35 were compared by paired-samples *t* test to the Pos 1

*Pos 1* postoperative day 1, *Pos 7* postoperative day 7, *Pos 35* postoperative day 35

*P < 0.05

## Discussion

M3M extraction is one of the more difficult operations in alveolar surgery, a difficulty directly related to the impacted M3M depth. The deeper the impacted M3M is, the worse the surgical field. Endoscopy can locally magnify the surgical field from various angles and help distinguish the tooth or bone position clearly. It can also help reduce the removal of adjacent bone and decrease hemorrhage during the surgery making the procedure less invasive. Engelke et al. found that endoscopy-assisted removal of M3M benefited the maintenance of surrounding bone structures and reduced surgical trauma [6, 7]. Our results of postoperative clinical assessment also confirmed the same conclusion. Juodzbalys et al. concluded that the endoscope could be an adjunct tool to assess the extraction socket morphology and bone condition without flap elevation [22]. Other scholars used endoscopy to observe and evaluate the implant position, conductive to improve implant site accuracy [9, 23, 24]. Endoscopy is valuable for exposed nerve observation after tooth extraction, helping IANI assessment. To date, no correlational study has analyzed the IAN under endoscopy. The advantage of endoscopy also included recording and analyzing the surgical procedures and images after surgery.

Previous studies on the IAN mainly used imaging techniques, lacking of direct observation and recording of the injured IAN. Several studies assessed the IAN using magnetic resonance imaging (MRI) to determine the extent of IANI after surgery and the lower lip numbness prognosis. Kotaki et al. performed a quantitative evaluation and fiber tracking of the normal IAN using diffusion tensor imaging (DTI) [25]. In our study, the exposure condition of the IAN, epineurium integrity, nerve fibers continuity and neurovascular bundle pulsation were observed by endoscopy. This technique might provide favorable conditions for IANI analysis and treatment in future.

Most studies use preoperative images, such as panoramic radiographs and CBCT, to judge the relationship between the M3M and the IAN. However, there are almost no studies using direct observations of the IAN [26]. Our result showed that the IAN was exposed with an average length of 4.65 mm and width of 1.89 mm, values significantly smaller than the measurements on the CBCT images. And there were five cases with no exposed IAN under endoscopy, suggesting that a thin bone layer surrounded the IAN when it was found to contact the tooth root on the CBCT images. In these cases, the risk of IANI should be lower than when the IAN is exposed. Therefore, physicians should be cautious when using CBCT to assess the region preoperatively. Seiko et al. found that a lingual or inter-radicular IAN position with respect to the M3M and multiple roots with perforated IAN increased the risk of IANI [27]. Susarla et al. found that the nerve injury rate increased by 20% if the IAN exposure was larger than 3 mm on the maxillofacial CT images [28]. Our study used endoscopy to record the IAN exposure more accurately and realistically than the naked eyes.

We found that the neurovascular bundle pulsation could be observed by endoscopy in nearly half of the cases helping determine whether the inferior alveolar artery was damaged delivering a high reference value for preventing intraoperative and postoperative bleeding. Therefore, endoscopy applied to observing and recording the damaged IAN could be beneficial as a reference for future treatment and prognosis of IANIs.

The M3M roots are usually closely associated with the IAN. It is easy to cause IANIs when removing M3Ms that contact, hook, or surround with the nerve. We used endoscopy-assisted dual-power system to remove the M3Ms. The contra-angle high-speed turbine handpiece was mainly used to eliminate the proximal crown resistance. The use of piezosurgery under real-time endoscopic visualization could largely remove the root resistance to avoid pulling or extruding the IAN during root extraction, and reduce the damage to the soft tissue [29]. The advantage of the piezosurgery was tissue selectivity, allowing cutting only at the level of bone. Besides, the working tips of piezo are much longer than the burs used for the handpieces. Therefore, they could be used for segmenting the roots in the deep site of mandible without blocking the field of view. Meanwhile, the tips used for piezo was wavy blade, which improved the efficiency of segmenting the roots. Under real-time endoscopic visualization, piezo could be used to remove bone resistance precisely, reduce unnecessary bone removal and reduce damage to IAN. But the working tips of piezo are easier to break than burs for handpieces, so it needs attention in use.

All our patients were at a high risk of nerve injury [27], but only two of the 30 patients whose IAN was observed under endoscopy complained of lip numbness. Our results showed a paresthesia rate 6.7% lower than the reported rate in the study by Susarla et al. possibly because we sighted the IAN during surgery [11]. Queral-Godoy et al. concluded that most cases of injured IAN would recover within six months, though in some cases recovery takes more than one year which likely to be associated with permanent damages [30]. Therefore, it can be suggested that the cause of numbness was traction or pressure from edema. The degree of nerve demyelination was so minimal that the recovery time was very short. This indicated that endoscopic operation combined with a dual-power system could avoid nerve injury effectively. Although piezosurgery could prevent damage to soft tissue, it could not completely avoid the nerve injury [31]. Therefore, there were two patients with lower lip numbness after surgery. Both the patients recovered in a very short time, which indicated the IANI caused by piezosurgery was mild.

QST is a sensitive way to detect somatosensory abnormalities such as lower lip numbness. This evaluation method quantitatively evaluates the subject responses based on various quantitative stimulus inputs (e.g., temperature, mechanical, electrical, and chemical stimulations) that act on various tissues (e.g., skin, muscle, viscera) and use various psychophysiological methods (e.g., threshold values, stimulus–response functions) [32]. QST can accurately assess sensory disorders by measuring thresholds and quantifying mechanical, temperature, and vibrational senses. QST sensitivity and specificity have been systematically studied, supporting its important role in diagnosing, classifying, long-term follow-up and evaluating neural diseases. Its protocol has been widely accepted in nerve function assessment and pain research [32, 33]. In this study, we measured QST parameters on the healthy and affected sides at all follow-up sessions using the data from the healthy side as self-control. Previous studies did not follow up on changes in the QST data of the healthy side after tooth extraction [16, 17, 32]. We found that these data changed but remained within the normal range. These variations may have been caused by the patient’s psychological reactions. Therefore, we used Z transformation to control the inference factors. Our results showed that all the Z-transformed values were fluctuated within the normal range between − 1.96 and 1.96. There were no significant differences comparing the preoperative and postoperative values, indicating that impacted M3M extraction by endoscopy-assisted, dual-power system did not change neurophysiologic pathways of IAN. WDT transmitted through unmyelinated C nerve fibers and showed the highest positive correlation with subjective symptoms a key factor in developing permanent subjective paresthesia [33, 34]. Once injured, recovery of the WDT speed would be the slowest among all QST parameters [35]. There was no significant difference in WDT between preoperative and one day after surgery, indicating no intraoperative damage to IAN. However, our results showed that there were significant differences between seven or 35 days after surgery and one day after surgery. This may be due to the increased threshold with multiple measurements, which was consistent with Juhl’s study. It had been reported that the healthy side endured a lower temperature before pain was experienced than at the baseline and heat pain thresholds were significantly higher during the observation period compared to the baseline values [36]. And the variation of HPT showed the same conclusion. There were no statistical differences in other QST parameters, because of little surgical trauma on IAN. We analyzed the correlation between the range of exposed IAN and postoperative QST parameters. Our results showed that TSL, MDT and CPT on one day after surgery were strongly correlated with the length of the exposed IAN and MDT was also strongly correlated with its width. However, this association disappeared on subsequent follow-up assessments. Almost no participant complained of subjective paresthesia. Besides TSL, MDT and CPT are sensitive to nerve injury. The observed correlation may be related to the temporary irritation caused by the surgery, which was mild and did not cause numbness in the patient's lower lip. Therefore, QST is a sensitive way to detect somatosensory abnormalities. TSL, MDT, and CPT could predict the range of the IAN exposed during surgery. The greater the change in these parameters, the greater possibly the impact on the nerve. Besides, two patients complained of lip numbness after the surgery, and TSL and WDT showed abnormal values in only one of them. This indicated that QST may be influenced by subjective responses and cannot completely represent the degree of IANI [34].

In conclusion, our study showed that the intraoperative exposed IAN endoscopic measurements were smaller than by CBCT and strongly correlated with TSL, MDT, and CPT on one day after surgery. Endoscopic observation and measurement of the IAN could benefit future diagnosis and treatment of IANI. Although the sample of our study was relatively small, it was found that not all exposed IAN in CBCT images were real exposure after surgery, which had clinical significance. Subsequent results of large samples will be presented in the further report.

## Data Availability

The data that support the findings of this study are available on request from the corresponding author. The data are not publicly available due to privacy or ethical restriction.

## References

[1] Farish SE, Bouloux GF (2007). General technique of third molar removal. Oral Maxillofac Surg Clin N Am.

[2] Marciani RD (2007). Third molar removal: an overview of indications, imaging, evaluation, and assessment of risk. Oral Maxillofac Surg Clin N Am.

[3] Moreno-Vicente J, Schiavone-Mussano R, Clemente-Salas E, Marí-Roig A, Jané-Salas E, López-López J (2015). Coronectomy versus surgical removal of the lower third molars with a high risk of injury to the inferior alveolar nerve. A bibliographical review. Med Oral Patol Oral Cir Bucal.

[4] Carvalho RWF, do Egito Vasconcelos BC (2011). Assessment of factors associated with surgical difficulty during removal of impacted lower third molars. J Oral Maxillofac Surg.

[5] Kang F, Sah MK, Fei G (2020). Determining the risk relationship associated with inferior alveolar nerve injury following removal of mandibular third molar teeth: a systematic review. J Stomatol Oral Maxillofac Surg.

[6] Engelke W, Fuentes R, Beltrán V (2013). Endoscopically assisted removal of a lingually displaced third molar adjacent to the inferior alveolar nerve. J Craniofac Surg.

[7] Engelke W, Beltrán V, Cantín M, Choi EJ, Navarro P, Fuentes R (2014). Removal of impacted mandibular third molars using an inward fragmentation technique (IFT)–method and first results. J Craniomaxillofac Surg.

[8] Huang ZQ, Huang ZX, Wang YY, Hu WJ, Fan S, Zhang DM, Chen WL (2015). Removal of the residual roots of mandibular wisdom teeth in the lingual space of the mandible via endoscopy. Int J Oral Max Surg.

[9] Beltrán V, Fuentes R, Engelke W (2012). Endoscopic visualization of anatomic structures as a support tool in oral surgery and implantology. J Oral Maxillofac Surg.

[10] Tay A, Go WS (2004). Effect of exposed inferior alveolar neurovascular bundle during surgical removal of impacted lower third molars. J Oral Maxillofac Surg.

[11] Weckx A, Agbaje JO, Yi S, Jacobs R, Politis C (2015). Visualization techniques of the inferior alveolar nerve (IAN): a narrative review. Surg Radiol Anat.

[12] Pavlíková G, Foltán R, Horká M, Hanzelka T, Borunská H, Šedý J (2011). Piezosurgery in oral and maxillofacial surgery. Int J Oral Maxillofac Surg.

[13] Tc A, Tu B, Hny C, Bg D (2021). Postoperative evaluation of Er:YAG laser, piezosurgery, and rotary systems used for osteotomy in mandibular third-molar extractions. J Oral Maxillofac Surg.

[14] Cicciù M, Stacchi C, Fiorillo L, Cervino G, Lenarda RD (2020). Piezoelectric bone surgery for impacted lower third molar extraction compared with conventional rotary instruments: a systematic review, meta-analysis, and trial sequential analysis. Int J Oral Max Surg.

[15] Rolke R, Magerl W, Campbell KA, Schalber C, Caspari S, Birklein F, Treede RD (2006). Quantitative sensory testing: a comprehensive protocol for clinical trials. Eur J Pain.

[16] Wang Y, Mo X, Zhang J, Fan Y, Wang K, Peter S (2018). Quantitative sensory testing (QST) in the orofacial region of healthy Chinese: influence of site, gender and age. Acta Odontol Scand.

[17] Yan ZY, Yan XY, Guo CB, Xie QF, Yang GJ, Cui NH (2020). Somatosensory changes in Chinese patients after coronectomy vs. total extraction of mandibular third molar: a prospective study. Clin Oral Invest.

[18] Kumar PP, Manjula S (2018). Comparative study of primary and secondary closure of the surgical wound after removal of impacted mandibular third molars. Oral Maxillofac Surg.

[19] Rolke R, Baron R, Maier C, Tölle TR, Treede RD, Beyer A, Binder A, Birbaumer N, Birklein F, Bötefür IC, Braune S, Flor H, Huge V, Klug R, Landwehrmeyer GB, Magerl W, Maihöfner C, Rolko C, Schaub C, Scherens A, Sprenger T, Valet M, Wasserka B (2006). Quantitative sensory testing in the German research network on neuropathic pain (DFNS): standardized protocol and reference values. Pain.

[20] Yang G, Baad-Hansen L, Wang K, Xie QF, Svensson P (2014). A study on variability of quantitative sensory testing in healthy participants and painful temporomandibular disorder patients. Somatosens Mot Res.

[21] Hartmann A, Welte-Jzyk C, Seiler M, Daubländer M (2017). Neurophysiological changes associated with implant placement. Clin Oral Implants Res.

[22] Juodzbalys G, Bojarskas S, Kubilius R, Wang HL (2008). Using the support immersion endoscope for socket assessment. J Periodontol.

[23] Engelke W, Lazzarini M, Stühmer W, Beltrán V (2015). Support immersion endoscopy in post-extraction alveolar bone chambers: a new window for microscopic bone imaging in vivo. PLoS ONE.

[24] Nahlieli O, Moshonov J, Zagury A, Michaeli E, Casap N (2011). Endoscopic approach to dental implantology. J Oral Maxillofac Surg.

[25] Kotaki S, Sakamoto J, Kretapirom K, Supak N, Sumi Y, Kurabayashi T (2016). Diffusion tensor imaging of the inferior alveolar nerve using 3T MRI: a study for quantitative evaluation and fibre tracking. Dentomaxillofac Rad.

[26] Cespedes-Sanchez JM, Ayuso-Montero R, Marí-Roig A, Arranz-Obispo C, López-López J (2014). The importance of a good evaluation in order to prevent oral nerve injuries: a review. Acta Odontol Scand.

[27] Kubota S, Imai T, Nakazawa M, Uzawa N (2020). Risk stratification against inferior alveolar nerve injury after lower third molar extraction by scoring on cone-beam computed tomography image. Odontology.

[28] Susarla SM, Sidhu HK, Avery LL, Dodson TB (2010). Does computed tomographic assessment of inferior alveolar canal cortical integrity predict nerve exposure during third molar surgery?. J Oral Maxillofac Surg.

[29] Basheer SA, Govind RJ, Daniel A, Sam G, Adarsh VJ, Rao A (2017). Comparative study of piezoelectric and rotary osteotomy technique for third molar impaction. J Contemp Dent Pract.

[30] Queral-Godoy E, Valmaseda-Castellón E, Berini-Aytés L, Gay-Escoda C (2005). Incidence and evolution of inferior alveolar nerve lesions following lower third molar extraction. Oral Surg Oral Med Oral Pathol Oral Radiol.

[31] Schaeren S, Jaquiéry C, Heberer M, Tolnay M, Vercellotti T, Martin I (2008). Assessment of nerve damage using a novel ultrasonic device for bone cutting. J Oral Maxillofac Surg.

[32] Jääskeläinen SK, Teerijoki-Oksa T, Virtanen A, Tenovuo O, Forssell H (2004). Sensory regeneration following intraoperatively verified trigeminal nerve injury. Neurology.

[33] Kim HK, Kim KS, Kim ME (2017). Thermal perception as a key factor for assessing effects of trigeminal nerve injury. J Oral Facial Pain Headache.

[34] Jensen TS, Baron R (2003). Translation of symptoms and signs into mechanisms in neuropathic pain. Pain.

[35] Freeman R, Chase KP, Risk MR (2003). Quantitative sensory testing cannot differentiate simulated sensory loss from sensory neuropathy. Neurology.

[36] Juhl GI, Svensson P, Norholt SE, Jensen TS (2006). Long-lasting mechanical sensitization following third molar surgery. J Orofac Pain.

